# Behavioral and Neuroimaging Evidence for Facial Emotion Recognition in Elderly Korean Adults with Mild Cognitive Impairment, Alzheimer’s Disease, and Frontotemporal Dementia

**DOI:** 10.3389/fnagi.2017.00389

**Published:** 2017-11-30

**Authors:** Soowon Park, Taehoon Kim, Seong A Shin, Yu Kyeong Kim, Bo Kyung Sohn, Hyeon-Ju Park, Jung-Hae Youn, Jun-Young Lee

**Affiliations:** ^1^Department of Education, Sejong University, Seoul, South Korea; ^2^Department of Psychiatry, Neuroscience Research Institute, Seoul National University and SMG-SNU Boramae Medical Center, Seoul, South Korea; ^3^Department of Biomedical Sciences, Seoul National University, Seoul, South Korea; ^4^Department of Nuclear Medicine, SMG-SNU Boramae Medical Center, Seoul, South Korea; ^5^Department of Psychiatry, Inje Univiersity Sanggye Paik Hospital, Seoul, South Korea; ^6^Graduate School of Clinical Counseling Psychology, CHA University, Pocheon, South Korea

**Keywords:** facial emotion recognition, mild cognitive impairment, Alzheimer’s disease, frontotemporal dementia, voxel-based morphometry

## Abstract

**Background:** Facial emotion recognition (FER) is impaired in individuals with frontotemporal dementia (FTD) and Alzheimer’s disease (AD) when compared to healthy older adults. Since deficits in emotion recognition are closely related to caregiver burden or social interactions, researchers have fundamental interest in FER performance in patients with dementia.

**Purpose:** The purpose of this study was to identify the performance profiles of six facial emotions (i.e., fear, anger, disgust, sadness, surprise, and happiness) and neutral faces measured among Korean healthy control (HCs), and those with mild cognitive impairment (MCI), AD, and FTD. Additionally, the neuroanatomical correlates of facial emotions were investigated.

**Methods:** A total of 110 (33 HC, 32 MCI, 32 AD, 13 FTD) older adult participants were recruited from two different medical centers in metropolitan areas of South Korea. These individuals underwent an FER test that was used to assess the recognition of emotions or absence of emotion (neutral) in 35 facial stimuli. Repeated measures two-way analyses of variance were used to examine the distinct profiles of emotional recognition among the four groups. We also performed brain imaging and voxel-based morphometry (VBM) on the participants to examine the associations between FER scores and gray matter volume.

**Results:** The mean score of negative emotion recognition (i.e., fear, anger, disgust, and sadness) clearly discriminated FTD participants from individuals with MCI and AD and HC [*F*(3,106) = 10.829, *p* < 0.001, η^2^ = 0.235], whereas the mean score of positive emotion recognition (i.e., surprise and happiness) did not. A VBM analysis showed negative emotions were correlated with gray matter volume of anterior temporal regions, whereas positive emotions were related to gray matter volume of fronto-parietal regions.

**Conclusion:** Impairment of negative FER in patients with FTD is cross-cultural. The discrete neural correlates of FER indicate that emotional recognition processing is a multi-modal system in the brain. Focusing on the negative emotion recognition is a more effective way to discriminate healthy aging, MCI, and AD from FTD in older Korean adults.

## Introduction

The study of social cognition indicates serial information processing about others’ behaviors or thinking in social situations. Recognition of others’ emotions, understanding of others’ mental state, and attributional orientations in explaining social situations are presented as the three domains of social cognition (e.g., Harvey and Penn, 2009). Among these social cognitive domains, how well individuals identify and recognize others’ emotions is the most popular research topic, and it reflects human instinctive function (Ekman and Friesen, 1971; Ekman, 1999; Elfenbein and Ambady, 2002). Accurate emotional recognition is certainly the prerequisite for development and maintenance of successful social interactions (Ruffman et al., 2008). If there is a deficit in the ability to identify others’ facial emotions, it causes many interpersonal difficulties such as miscommunication with others and low social competency, and more general difficulties such as lower personal well-being and greater depression (Carton et al., 1999). Therefore, there is great interest among researchers in understanding the mechanisms of and influences on facial emotion recognition (FER) in humans.

Basic facial emotions (i.e., anger, fear, disgust, sadness, happiness, and surprise), which humans use to convey emotions, are considered to be innate and cross-cultural (Ekman and Friesen, 1971). The classical study by Ekman and Friesen (1971) supports the presence of universal roles for FER in social networks. There are three theories regarding emotion processing, namely, the limbic system model, the right hemisphere model, and the multimodal system model (Kumfor and Piguet, 2012). These theories are basically grounded in neuroanatomy. Initially, a single system model for emotion (i.e., limbic system theory of emotion) was the most widely accepted early theory of emotion (Maclean, 1952). However, critics argued that the term “limbic system” does not consistently define specific brain regions. In addition, there are other brain regions (e.g., orbitofrontal cortex and ventromedial prefrontal cortex) that are thought to have critical roles in emotion processing, but are not involved in the limbic system (Pessoa, 2008). The right hemisphere model suggests that there is a strong lateralization of emotion processing toward the right hemisphere. Even though many previous studies have demonstrated that right-side lesions are critical to the processing of emotions (e.g., Borod et al., 1998; Perry et al., 2001), this model cannot fully explain emotional recognition deficits in patients with left-side atrophy (e.g., Calabria et al., 2009). Ekman (1999) has proposed the multi-system model of emotion, which states that unique patterns of neural mechanisms trigger each emotion. In other words, discrete neural substrates are responsible for specific emotion recognition deficits. Therefore, it is possible that processes involved in the identification of facial emotions vary based on the content of the emotion (Posamentier and Abdi, 2003). Previous studies support the multimodal system model (Phan et al., 2002; Kumfor et al., 2014). The current study was also performed based on the framework of the multimodal theory.

A common and effective approach to understanding human FER is to examine patients who show a deficit in FER. Patients with frontotemporal dementia (FTD) show changes in interpersonal behaviors such as apathy, social misconduct, and impaired social awareness (Adenzato et al., 2010; Henry et al., 2014). FTD is a term encompassing three subtypes of FTD: behavioral variant FTD (bvFTD), semantic dementia, and progressive non-fluent aphasia. FTD classification is performed based on the patients’ dominant clinical symptoms. Patients with bvFTD have disturbances in behaviors, and those with semantic dementia or progressive non-fluent aphasia have fluent or non-fluent disturbances in language. Patients with bvFTD and semantic dementia have deficits in emotional processing (for a review, see Kumfor and Piguet, 2012). Patients with frontal or behavioral variants of FTD in particular have deficits in processing fear, anger, disgust, and sadness when compared to healthy controls (Lough et al., 2006), and additionally are impaired in both facial and vocal emotion recognition (Keane et al., 2002; Shany-Ur and Rankin, 2011).

A recent meta-analysis study analyzed 19 studies involving 329 healthy controls (HCs), 162 patients with bvFTD, and 147 patients with Alzheimer’s disease (AD) (Bora et al., 2016). The authors found that patients with bvFTD had significant deficits in both negative and positive FER when compared to HCs. Furthermore, the effect sizes were larger for negative emotions (e.g., anger = 1.48, disgust = 1.41) than for positive emotions (e.g., happy = 0.60). Patients with bvFTD also had impaired recognition of all emotions except for happiness (i.e., anger, disgust, fear, sadness, and surprise) when compared to those with AD. The effect size was largest for disgust (i.e., 1.05). These results indicate that there are distinct FER deficits depending on both the patient diagnosis and the contents of the emotions. Therefore, specific performances for each emotion and the diagnostic status should be clarified. Examination of FER performance and the neural characteristics of patients with FTD, as well as individuals in other groups (e.g., healthy older adults and patients with AD) would provide us with a fundamental understanding of the behavioral characteristics and neural networks involved in the preservation of or deficits in FER.

Patients with AD and those with mild cognitive impairment (MCI) are also known to have poor FER, yet patients with MCI typically do not display the severe problematic behaviors seen in the early stage of MCI (McCade et al., 2012). Both patients with FTD and those with AD show lower scores in the Ekman 60 task than those shown by HC (Miller et al., 2012). AD and MCI patients also show a low ability to attribute others’ mental states, emotional understanding, and emotional regulation (for a review, see Kemp et al., 2012). Previous studies indicate that only patients with the amnestic subtype of MCI, but not those with non-amnestic MCI, have emotional recognition deficits (McCade et al., 2013b).

Determining the distinct FER profiles in HCs and patients with dementia, including those with amnestic MCI, AD, or FTD, would help us to find specific emotions that would benefit from interventions at certain disease progression stages. Previous studies have compared FER performance in HCs and patients with MCI, AD, and FTD. Bediou et al. (2009) investigated social cognitive functions (i.e., FER and eye gaze direction determination) in 10 amnestic MCI patients, 10 patients with mild dementia related to AD, 10 patients with FTD, and 10 HC. Results showed only FTD was impaired in FER compared to HC and mild dementia related to AD in the higher intensity (i.e., more than 80%) of facial emotions (Bediou et al., 2009). However, this study focused on the distinct performance in dementia groups depending on the intensity of facial emotions, and not estimating the distinct processing of different emotional content.

Rosen et al. (2006) investigated performance of facial expression recognition, measured by the Florida affect battery, in 5 HC, and 15 AD, 1 MCI, 25 FTD, and 4 progressive supranuclear palsy patients (Rosen et al., 2006). Here, patients with FTD include those with bvFTD, semantic dementia, and progressive non-fluent aphasia. This study revealed the neuroanatomical correlates of impaired FER, which is the region of right lateral inferior temporal gyrus and right middle temporal gyrus (BA 21) was correlated with accuracy of negative FER. However, the sample size was small and only four emotions (i.e., fear, anger, sadness, and happiness) were considered. No profiles of performance for each emotion and each group were reported.

Furthermore, a few studies have reported that FER performance is dependent on the contents of emotion, although the results of these studies are inconsistent (Lough et al., 2006; Kumfor and Piguet, 2012). In particular, patients with FTD (i.e., four patients with the frontal variant, three with the temporal variant, and three with a mixed frontotemporal atrophy pattern) were found to have impaired recognition of anger and surprise, while the recognition of disgust, happiness, sadness, and fear was the same as that shown by controls (Kessels et al., 2007). Patients with FTD patients also had impaired recognition of all facial emotions (i.e., anger, fear, disgust, sadness, happiness, and surprise) when compared to healthy controls in studies by Diehl-Schmid et al. (2007) and Snowden et al. (2008).

Recognition of disgust is only preserved in patients with AD when compared to older and younger HCs (Henry et al., 2008). Performances of emotion recognition in MCI is varied (for a review, see McCade et al., 2012). This inconsistency may be a result of the difference in the characteristics of participants (e.g., age, gender, subtype of dementia, or cognitive status), different tasks (e.g., types of stimuli or procedures used for the tasks), or different stimulus intensities (Elferink et al., 2015). Therefore, more studies of the participants’ characteristics or tasks are needed. Finding differences in the performance of FER based on emotional content may provide evidence regarding the types of emotions that are more affected in dementia. It is crucial to examine the time at which these deficits actually start to appear, as it would allow us to understand impairments in emotion recognition as continuative dementia trajectories.

In addition, if distinct neural substrates are used to interpret different emotional expressions and patients with dementia, such as those with FTD and AD show degeneration in specific brain regions, the sensitivity of detecting dementia type may be improved depending on recognition of each emotion. One study compared emotional recognition ability in FER between patients with FTD and HCs (Kumfor et al., 2013). However, there has been a lack of interest in finding the profiles of different emotions and determining emotions that have the largest effect sizes in distinguishing patients with FTD from HCs, and patients with MCI or AD. Due to the many competing demands and time constraints, clinicians need to prioritize their efforts by implementing screening tools that are evidence-based and both time- and cost-effective.

We address the important gap in the literature related to the participants included and the methodological approaches (i.e., brain imaging). We simultaneously considered HC, MCI, AD, and FTD participants in order to understand the distinct functioning of emotion recognition processing in the neural system, depending on emotional content. Furthermore, it is ecologically more valid to identify facial emotions of Korean actors, as Korea has a very ethnically homogenous population. Older Koreans thus generally see and meet Korean people in their everyday lives. A previous study has revealed that individuals more accurately recognize emotions of those in the same ethnicity or regional group (Elfenbein and Ambady, 2002). While recognition of emotion is considered as a universal function, evidence that FER is cross-cultural should be examined. Based on previous studies examining FER in HCs and patients with FTD, AD, and MCI, we hypothesized that patients with FTD would have lower FER performance than HCs or patients with MCI. Patients with AD are also expected to have deficits in FER (Hsieh et al., 2013). However, we hypothesized that the difference between patients with FTD and those with AD would be equivocal, as there are inconsistent results in this regard for the different emotions. Furthermore, we hypothesized that negative emotional content would have a larger effect size when distinguishing among groups than positive emotional content (Bora et al., 2016).

Based on the multi-modal perspectives on emotional processing, the purpose of this study was to (1) identify the different profiles of FER of specific emotions in HC, MCI, AD, and FTD, and (2) find brain structures that are closely associated with FER ability. Through this approach, we combined behavioral performance with brain imaging data. A Korean version of the FER test would be useful when testing the severity of emotion recognition deficits in Korea. Here, we present basic information regarding the performance of older adults on this test.

## Materials and Methods

### Development of the Facial Emotion Recognition Test

In the present study, we developed a Korean version of the FER test based on Ekman’s 60 faces (Ekman and Friesen, 1976). Professional Korean actors acted out six emotions and presented neutral faces. The Ekman 60 faces test has been most widely used in facial expression studies; this previous test (i.e., the Ekman 60 faces test) utilized a series of 60 photographs, which are presented to participants, to assess the ability to recognize basic facial emotions. The FER used in the current study was adopted using the same administration approaches used for the Ekman 60 faces test, although the FER test used 35 facial stimuli. This small number of stimuli would be helpful to relieve fatigue, anxiety, and frustration of participants and researchers. In addition, it would help to reduce the time and cost of disease diagnosis in patients.

More specifically, the FER test used in the current study was developed based on the Social cognition and Emotional Assessment (SEA) (Funkiewiez et al., 2012) which uses the Pictures of Facial Affect set, developed by Ekman and Friesen (1976). Facial emotions indicating fear, anger, disgust, sadness, surprise, happiness, and neutral face were expressed by four professional actors, and grayscale pictures of the emotions were taken following the instructions of Ekman. Ten pictures of each emotion were provided to 40 graduate students in the Department of Psychology so that they could assess and select the proper stimuli to be used. The intensity of each emotion was defined as the extent to which the expression of each emotion. Therefore, the intensity of each facial emotion stimulus was measured on a 10-point Likert-type scale (1 = very weak expression, 10 = very strong expression). The pictures were presented randomly to control for the order effect. A total of 35 pictures (5 pictures for the six emotions and the neutral faces) with the strongest emotional intensity were selected from those with inter-rater consistencies higher than 0.70. The pictures labeled as “neutral” were selected based on inter-rater consistency, as the intensity of the neutral face could not be measured on a scale. The mean intensities of the emotions were as follows: fear = 6.36 (*SD* = 1.39), anger = 6.37 (*SD* = 1.56), disgust = 6.22 (*SD* = 1.57), sadness = 5.90 (*SD* = 1.66), surprise = 7.18 (*SD* = 1.53), and happiness = 6.13 (*SD* = 1.41). The repeated measure ANOVA showed that there was significant difference in the intensities among the contents (Wilks’ Lambda = 0.603, *F* = 4.616, *p* = 0.002, $\eta_{p}^{2}$ = 0.397). Follow-up pairwise comparisons with Bonferroni adjustment showed intensity of surprise was higher than others; fear (*p* = 0.061, which was marginally significant), anger (*p* = 0.042), disgust (*p* = 0.030), sadness (*p* < 0.001), and happiness (*p* < 0.001).

### Participants

A total of 127 older adults were recruited, although 17 were excluded in the data analysis. We were thus left with 110 participants. The exclusion criteria for participation in the study were as follows: any physical illness or history of neurological disease, except dementia, that may affect cognitive skills and perception, auditory or visual difficulties that could disrupt the test procedure, impaired physical mobility that might influence each process, refusal to give consent, no education, and inability to properly complete the test as judged by an examiner. Based on these exclusion criteria, two subjects were excluded because they were unable to normally perceive stimuli and six were excluded who had a history of neurological disease. In addition, eight older adults who had never had formal education and one participant who did not complete the tests because of private reason were excluded. As a result, 110 older adults (33 HCs and 32 patients with MCI, 32 with AD, and 13 with FTD) participated in the study.

The participants were classified into the groups of HC, MCI, AD, and FTD according to the criteria of DSM-IV (The Diagnostic and Statistical Manual of Mental Disorders, Fourth Edition), the criteria of the National Institute of Neurological and Communicative Disorders and Stroke and the AD and Related Disorders Association (NINCDS-ADRDA) (Mckhann et al., 1984), Petersen’s criteria (Petersen et al., 1997), and Neary Criteria (Neary et al., 2005). The patients had the amnestic subtype of the disease. The FTD group contained eight patients with bvFTD and five with semantic dementia. Of these 110 participants, the brain magnetic resonance imaging (MRI) scans of 93 (27 HC, 30 MCI, 27 AD, 9 FTD) were collected.

### Neuropsychological Assessments

The Mini-Mental State Examination (MMSE), the Clinical Dementia Rating (CDR), and the Consortium to Establish a Registry for Alzheimer’s Disease (CERAD) neuropsychological battery (Lee J.H. et al., 2002) were administered to test the cognitive ability of the participants.

#### MMSE

The MMSE is a test developed to screen for impairments in neurocognitive aspects, and can be administered in 5–10 min (Folstein et al., 1975). The scores range from 0 to 30, with higher scores indicating better cognition. Scores below 25 suggest the presence of cognitive impairment. The Korean version of the MMSE was developed for elderly populations as a part of the CERAD packet (Lee J.H. et al., 2002), with measurements including judgment, attention, orientation, short-term memory, following verbal command, naming, and double-pentagon copying.

#### CDR

The CDR is a measurement used to assess dementia severity, and was developed by Hughes et al. (1982) and revised by Morris (1993). The Korean version of the test was developed and validated by Choi et al. (2001). The test results in a global composite score used to assess the severity of dementia based on scores in six areas: memory, orientation, judgment and problem solving, community affairs, home and hobbies, and personal care. The composite rating has five levels of CDR (0, 0.5, 1, 2, and 3), which are used to determine the intensity of dementia: none, questionable, mild, moderate, and severe, respectively.

#### CERAD

The CERAD neuropsychological battery for Koreans was developed by Lee J.H. et al. (2002) based on the original assessment batteries used to assess cognitive ability (Morris et al., 1993). The test facilitates the diagnosis of AD and contains several neuropsychological measurements: Verbal Fluency, Boston Naming Test, Mini-Mental State Examination, Word List Memory, Constructional Praxis, Word List Recall, Word List Recognition, and Constructional Praxis Recall.

### Administration of the Facial Emotion Recognition Test

The FER test was carried out on a personal computer in an isolated room. Instructions were given in both verbal and visual form, and the participants were directed to answer verbally. The instructions were as follows:

“Some facial pictures are going to be shown from now on. Each picture expresses one of the emotions and you should indicate the kind of emotion that the picture is representing from the examples on the right side. Look at the example item; which of the following is indicated by the picture?”

The example facial item and the seven options were presented (**Supplementary Figure S1**). The participant was allowed to identify the emotion that was expressed by the facial stimulus. Another set of instructions for the main items was then given as the participants were deemed to understand how the test works. The list of six emotions (i.e., fear, anger, disgust, sadness, surprise, and happiness) and the neutral faces were sequentially provided on the monitor. Five facial pictures for each of the six emotion and five neutral faces (i.e., a total of 35 stimuli) were provided to the patients, along with the instructions, as the experiment began. The instructions were as follows:

“The actual test is going to be conducted from now on. Choose any example from the right side that indicates the given emotion. It will start as soon as you are ready.”

The stimuli were given once participants were fully aware of the instructions. Examples of pictorial stimuli are presented in **Supplementary Figure S2**. Each stimulus was displayed for a maximum of 7 s; the examiner moved on to the next stimulus when a verbal response was made. The instructor recorded the participant’s responses on the answer sheet. We planned to consider the response a wrong answer when the participants did not provide a response within 7 s, or when more than two options were verbally delivered. However, all participants chose one option within 7 s, so there were no missing data in the FER analysis. The sequence of emotions was completely randomized. 5–10 min were taken for the whole experiment.

### Sampling and Procedures

The participants were recruited from SMG-SNU Boramae Medical Center and Dongjak-Gu Center for Dementia from February 2013 to February of 2014. They visited these centers for prevention and diagnosis, and to receive medical care for cognitive decline. Among the visitors, older adults who consented to participate in the experiments were included in the study. The participants completed written consent that they voluntarily participated in the experiment and that they were fully informed of the specific details. The MMSE, CDR, and CERAD were conducted to measure cognitive function and daily discomfort of the participants. Neuropsychological assessments and the FER test were carried out in an isolated and quiet room by two trained graduate students majoring in counseling. This process occurred in the morning between 9 and 12 a.m. A total of 93 participants, who agreed to and were clinically able to get brain imaging, participated in the MRI imaging study. The participants were not provided with any reward or payment.

### Brain Imaging Analysis

Ninety-three participants (27 NC, 30 MCI, 27 AD, and 9 FTD) of the total recruited in this study underwent MRI (3 Tesla, Achieva, Philips Healthcare, Netherlands), and structural T1 was acquired. Image preprocessing steps for voxel-based morphometry using T1 images were performed using Statistical Parametric Mapping 8 implemented in Matlab (2014a, Mathworks^1^). The structural images were segmented into gray and white matter, and normalized into a standard space using DARTEL (diffeomorphic anatomical registration using exponentiated lie algebra) algorithms and tissue probability maps that are included in SPM 8 software. Then the images were modulated to preserve tissue volume after warping, and finally smoothed with an isotropic Gaussian kernel of 10 mm × 10 mm × 10 mm at full-width at half-maximum. First, gray matter volume changes were examined using *t*-tests between each patient group and the HC group in a voxel-wise manner. Age, years of education, and total intracranial volume were added as covariates of no interest (**Supplementary Figure S3**). For correlation analysis, a multiple regression model was employed for each emotion measure to identify regional correlates of FER in a voxel-wise manner; age, year of education, and total intracranial volume were covariates of no interest. The correlation analysis was carried out for HCs and patients with MCI, AD, and FTD in combination in order to examine the correlated brain structures in the overall participant population. The statistical thresholds were set at *p* < 0.001, uncorrected for multiple comparisons and minimal size of spatially continuous cluster greater than 50 voxels.

### Behavioral Data Analysis

Analysis was conducted using SPSS-PC software (version 18.0 for Windows, United States, IL). The total score of the FER test was the mean score for all seven categories (i.e., fear, anger, disgust, sadness, neutral, surprise, and happy). The maximum score was 5. The value of negative emotions was calculated as the mean of fear, anger, disgust, and sadness; thus, the maximum score was 5. The value of positive emotions was calculated as the mean of surprise and happy, thus the maximum score was 5.

Before the analysis of descriptive and inferential statistics, missing values and distributions of normality for each variable were checked. There were no missing values in all FER scores, thus no missing data technique was performed. In addition, the absolute values of skewness and kurtosis of all the variables was less than 1, suggesting the normality of scores was acceptable (Curran et al., 1996).

Descriptive statistics were used to describe participant characteristics. Sex and CDR distributions in the 4 groups were compared using the chi-square test. Mean age, education years, and neuropsychological scores (i.e., MMSE and CERAD) for each of the four groups were compared by one-way ANOVA (Analysis of Variance) with Tukey’s HSD *post hoc* tests.

For analysis of group differences in total scores, a one-way ANOVA with covariates of age and education was computed. Repeated measures two-way ANOVA, with covariates of age and education, was performed to establish whether there were significant differences in the recognition of negative and positive emotions among groups. Therefore, positive mean and negative mean scores were analyzed simultaneously in one repeated-measures ANOVA. Another repeated measures two-way ANOVA, with covariates of age and education, was carried out to establish whether there were significant differences in the profiles of each emotion among groups. In the repeated measures ANOVAs, participant responses (i.e., positive mean and negative mean; six emotions and neutral facial stimuli) were treated as within-subject variables. Group (i.e., HC, MCI, AD, and FTD) was included as a between-subjects variable. When the assumption of sphericity had been violated, degrees of freedom were corrected using Huynh-Feldt estimates of sphericity. Tukey’s HSD pairwise comparisons were used to reveal group differences in each emotion.

Receiver operating characteristics (ROC) curve analyses were performed to evaluate the discriminating power of the total, negative, and positive scores, and that of each emotion or the neutral faces to differentiate patients with FTD from those in the other groups (i.e., HC, MCI, and AD) using graphic methods. The area under the curve (AUC) was used as a measure of the overall performance of each ROC curve. We also assessed whether the AUC values were significantly different using a comparison of the ROC curves performed using MedCalc software (MedCalc Software bvba; Ostend, Belgium). Finally, optimal cut-off points for the scores (i.e., total, negative, positive, fear, anger, disgust, sadness, neutral, surprise, and happiness) were calculated by selecting the point on the ROC curve that maximized both sensitivity and specificity. Two-tailed *p*-values below 0.05 were considered statistically significant throughout the analysis.

### Ethics Statement

The review board of Boramae Medical Center, South Korea approved the study protocol and all participants gave written informed consent. The research was conducted according to the Helsinki Declaration guidelines.

## Results

### Demographic and Neuropsychological Characteristics

The age, education, gender, CDR, MMSE, and CERAD scores for each group (i.e., HC, MCI, AD, FTD) are presented in **Table 1**. The average age was 73.75 years (*SD* = 6.75), and the average duration of education was 10.59 years (*SD* = 3.94). Of the total participants, there were 41 males and 69 females. There were no significant differences in gender, but there were significant differences in age and education [*F*_age_(3,106) = 4.84, *p* = 0.003; *F*_edu_(3,106) = 3.24, *p* = 0.025]. A *post hoc* analysis revealed that the AD group had a higher mean age than the HC group, while the HC group had a longer duration of education than the AD group. Differences concerning clinical characteristics among the groups were also significant [χ^2^_CDR_ (6) = 121.14. *p* < 0.001, *F*_MMSE_(3,106) = 44.75, *p* < 0.001; *F*_CERAD_(3,103) = 31.24, *p* < 0.001]. The MMSE and CERAD scores were able to distinguish between HCs and patients with MCI and AD, but not between those with AD and those with FTD.

**Table 1:** Demographic and neuropsychological characteristics for each group.

	HC	MCI	AD	FTD	*F* or χ^2^	*p*	Total
	(*n* = 33)	(*n* = 32)	(*n* = 32)	(*n* = 13)			(*N* = 110)
**Demographics**							
Age	70.97 (6.45)^a^	74.34 (4.56)^ab^	76.75 (8.47)^b^	71.92 (3.64)^ab^	4.841	0.003	73.75 (6.75)
Education	12.27 (3.19)^a^	10.25 (3.43)^ab^	9.50 (3.94)^b^	9.85 (5.64)^ab^	3.240	0.025	10.59 (3.94)
Gender (M:F)	11:22	11:21	15:17	4:9	1.831	0.608	41:69
**Neuropsychological characteristics**							
CDR (0/0.5/1)	27/6/0	0/32/0	0/15/17	0/5/8	121.140	<0.001	27/58/25
MMSE	27.91 (1.96)^a^	24.69 (2.42)^b^	19.00 (3.42)^c^	18.62 (7.24)^c^	44.746	<0.001	23.28 (5.19)
CERAD	65.79 (12.38)^a^	55.91 (11.33)^b^	38.37 (9.85)^c^	40.75 (17.88)^c^	31.244	<0.001	52.43 (16.56)

*Mean (standard deviation). For the variables, means within a row with non-common superscripts were significantly different using Tukey’s HSD *post hoc* tests (*p* < 0.05). HC, healthy control; MCI, mild cognitive impairment; AD, Alzheimer disease; FTD, frontotemporal dementia; M, male; F, female; CDR, clinical dementia rating global; MMSE, Mini-Mental State Examination; CERAD, consortium to establish a registry for Alzheimer’s disease neuropsychological battery.*

### Facial Emotion Recognition across the Groups

**FIGURE 1:**
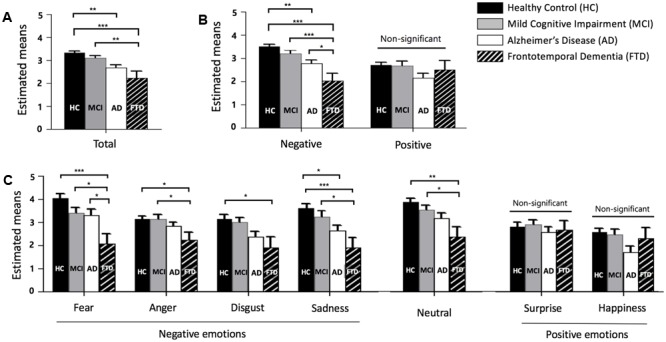
Facial emotion recognition scores across groups. **(A)** Total score (mean of fear, anger, disgust, sadness, neutral, surprise, and happiness). **(B)** Negative (mean of fear, anger, disgust, and sadness) and positive (mean of surprise and happiness) emotion. **(C)** Each emotion, error bars represent standard errors. HC = healthy control, MCI = mild cognitive impairment, AD = Alzheimer’s disease, and FTD = frontotemporal dementia. ^∗^*p* < 0.05, ^∗∗^*p* < 0.01, ^∗∗∗^*p* < 0.001.

Descriptive statistics, *post hoc* tests using the Tukey’s HSD, $\eta_{p}^{2}$, and observed power of total, negative, and positive scores, as well as that for each emotion, are presented in sequence in **Table 2**. **Figure 1** also shows the profiles of the emotions across groups in terms of (A) total, (B) negative and positive emotions, and (C) each emotion.

**Table 2:** Means and standard deviations of scores on the facial emotion recognition across groups.

	HC	MCI	AD	FTD	*F*	*p*	η^2^	Observed	Total
	(*n* = 33)	(*n* = 32)	(*n* = 32)	(*n* = 13)				power	(*N* = 110)
Total	3.32 (0.51)^a^	3.10 (0.64)^ab^	2.66 (0.78)^bc^	2.22 (1.09)^c^	8.040	<0.001	0.188	0.989	2.94 (0.79)
Negative	3.49 (0.59)^a^	3.20 (0.90)^ab^	2.80 (0.84)^b^	2.04 (1.14)^c^	10.829	<0.001	0.235	0.999	3.03 (0.94)
Positive	2.70 (0.72)^a^	2.69 (1.07)^a^	2.16 (1.06)^a^	2.50 (1.46)^a^	1.944	0.127	0.052	0.489	2.51 (1.04)
Fear	4.06 (1.14)^a^	3.41 (1.37)^a^	3.31 (1.45)^a^	2.08 (1.55)^b^	6.827	<0.001	0.162	0.973	3.42 (1.46)
Anger	3.15 (0.83)^a^	3.16 (1.14)^a^	2.84 (1.02)^ab^	2.23 (1.30)^b^	3.007	0.034	0.078	0.695	2.95 (1.07)
Disgust	3.15 (1.09)^a^	3.00 (1.11)^ab^	2.38 (1.36)^ab^	1.92 (1.71)^b^	4.334	0.006	0.109	0.857	2.74 (1.32)
Sadness	3.61 (1.09)^a^	3.25 (1.57)^a^	2.66 (1.18)^ab^	1.92 (1.50)^b^	6.317	0.001	0.152	0.962	3.03 (1.41)
Neutral	3.88 (0.93)^a^	3.53 (1.16)^a^	3.19 (1.28)^ab^	2.38 (1.56)^b^	5.430	0.002	0.133	0.929	3.40 (1.26)
Surprise	2.82 (1.01)^a^	2.91 (1.06)^a^	2.59 (1.21)^a^	2.69 (1.44)^a^	0.445	0.721	0.012	0.137	2.76 (1.13)
Happiness	2.58 (0.90)^a^	2.47 (1.46)^a^	1.72 (1.44)^a^	2.31 (1.70)^a^	2.602	0.056	0.069	0.624	2.26 (1.37)

*Mean (standard deviation). For the variables, means within a row with non-common superscripts were significantly different using Tukey’s HSD *post hoc* tests (*p* < 0.05). Total was the mean of all seven emotional scores; negative score was the mean of fear, anger, disgust, and sadness; positive score was the mean of surprise and happiness. HC, healthy control; MCI, mild cognitive impairment; AD, Alzheimer disease; FTD, frontotemporal dementia.*

#### Total Score Difference across Groups

A one-way ANOVA revealed group differences in total scores of FER [*F*(3,104) = 8.040, *p* < 0.001]. FTD patients showed significantly lower total scores than HC and MCI patients, but not AD patients. HCs had higher total scores than patients with AD.

#### Negative and Positive Score Differences across Groups

For the scores of negative and positive emotions, a significant interaction between the type of emotion and groups was observed [*F*(3,104) = 5.043, *p* = 0.003]. A univariate ANOVA showed differences between groups in negative emotion recognition [*F*(3,106) = 10.829, *p* < 0.001] but not for positive emotions [*F*(3,106) = 1.944, *p* = 0.127]. The negative emotion score showed an effect size of 0.235 and observed power of 0.999, while the positive emotion score showed an effect size of 0.052 and observed power of 0.489. Only negative emotion recognition distinguished FTD from HC, MCI, and AD.

#### Differences for Each Emotion across Groups

For the analysis of each emotion, the results show that there was a significant interaction between the type of emotion and dementia groups [*F*(16.462,570.694) = 2.073, *p* = 0.008]. This indicated that the profiles of each score were different for each dementia group. Among each emotion, fear showed the largest effect size (η^2^ = 0.162) and the largest observed power (0.973). Fear distinguished FTD from HC, MCI, and AD. Sadness and neutral showed large effect sizes of 0.152 and 0.133, respectively, and these emotions distinguished FTD from HC and MCI, but not AD. The *F*-values of anger and disgust were significant and these emotions were able to differentiate FTD from HC. Surprise and happiness showed no significant difference among groups (*p* = 0.721 for surprise, *p* = 0.056 for happiness).

### Receiver Operating Characteristic Curves of the Facial Emotion Recognition for the Detection of Frontotemporal Dementia

The ROC curves were plotted in order to determine the degree to which FER discriminated between HC, MCI, AD, and FTD groups. ROC area, cutoff scores, sensitivity, and specificity for the detection of FTD are presented in **Table 3**. The area under the curve (AUC) for negative emotions was high, while the AUC from positive emotions was relatively lower.

**Table 3:** Receiver operating characteristics (ROC) curves, cutoff points, sensitivity, and specificity of the FER for the discrimination between HC/FTD, MCI/FTD, and AD/FTD.

	HC (*n* = 33) vs. FTD (*n* = 13)					MCI (*n* = 32) vs. FTD (*n* = 13)					AD (*n* = 32) vs. FTD (*n* = 13)				
	AUC	*p*	Cutoff	Sen	Spe	AUC	*p*	Cutoff	Sen	Spe	AUC	*p*	Cutoff	Sen	Spe
Total	0.821	0.001	<2.642	0.692	0.909	0.744	0.011	<2.714	0.692	0.719	0.615	0.229	<2.071	0.538	0.781
Negative	0.881	<0.001	<2.875	0.846	0.848	0.770	0.005	<2.875	0.846	0.625	0.708	0.030	<2.625	0.615	0.656
Positive	0.610	0.252	<2.250	0.538	0.788	0.559	0.540	<2.250	0.538	0.594	0.424	0.430	<2.250	0.538	0.438
Fear	0.850	<0.001	<3.500	0.846	0.788	0.756	0.008	<2.500	0.615	0.844	0.728	0.017	<2.500	0.615	0.750
Anger	0.740	0.012	<2.500	0.692	0.788	0.728	0.017	<2.500	0.692	0.781	0.665	0.086	<2.500	0.692	0.656
Disgust	0.702	0.035	<2.500	0.615	0.727	0.681	0.059	<2.500	0.615	0.625	0.578	0.416	<2.500	0.615	0.500
Sadness	0.814	0.001	<2.500	0.692	0.848	0.730	0.017	<2.500	0.692	0.688	0.663	0.089	<2.500	0.692	0.500
Neutral	0.780	0.003	<2.500	0.615	0.909	0.716	0.024	<2.500	0.615	0.813	0.659	0.098	<2.500	0.615	0.719
Surprise	0.534	0.724	<2.500	0.462	0.636	0.547	0.625	<2.500	0.462	0.625	0.481	0.841	<2.500	0.462	0.500
Happiness	0.614	0.232	<2.500	0.769	0.515	0.536	0.707	<2.500	0.769	0.438	0.376	0.197	<2.500	0.769	0.250

*Total was the mean of all seven emotional scores; negative score was the mean of fear, anger, disgust, and sadness; positive score was the mean of surprise and happiness. HC, healthy control; FTD, frontotemporal dementia; MCI, mild cognitive impairment; AD, Alzheimer’s disease AUC; area under curve; Sen, sensitivity; Spe, specificity.*

**FIGURE 2:**
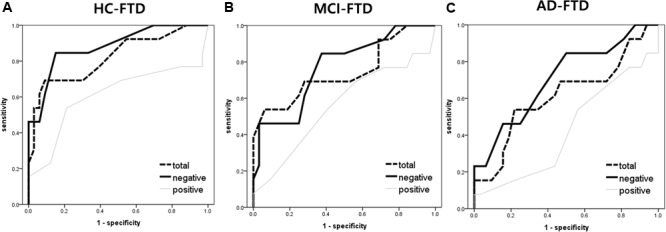
Receiver operating characteristic curve analysis of the facial emotion recognition scores (i.e., total, negative, and positive) for detecting frontotemporal dementia. **(A)** HC vs. FTD, **(B)** MCI vs. FTD, and **(C)** AD vs. FTD. Total = mean of all 7 (i.e., fear, anger, disgust, sadness, neutral, surprise, and happiness) emotional scores; negative score = mean of fear, anger, disgust, and sadness; positive score = mean of surprise and happiness.

**FIGURE 3:**
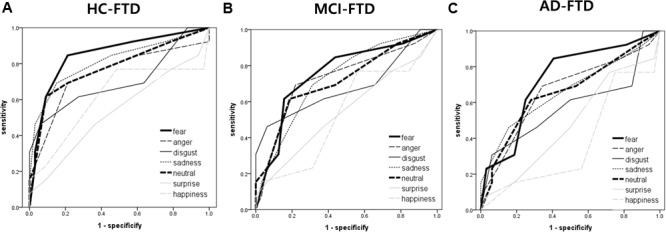
Receiver operating characteristic curve analysis of each emotion (i.e., fear, anger, disgust, sadness, neutral, surprise, and happiness) in facial emotion recognition for detecting frontotemporal dementia. **(A)** HC vs. FTD, **(B)** MCI vs. FTD, and **(C)** AD vs. FTD.

The AUC for FER of fear was the highest of all emotion analyses, discriminating FTD from other groups quite precisely (AUC > 0.70). Lower AUCs were demonstrated for surprise and happiness, ranging from 0.40 to 0.60 on average. **Figure 2** shows the ROC curves of grouped scores; total, negative, and positive. **Figure 3** shows the respective ROC curves of the test scores for each emotion.

The statistical significance of the difference between each AUC was also analyzed. When discriminating FTD from HC, negative emotions showed a significantly higher AUC than positive emotions (*z* = 2.245, *p* = 0.025). In the comparison of each emotion, fear showed a significantly higher AUC compared to surprise (*z* = 2.443, *p* = 0.015), and a marginally higher AUC than happiness (*z* = 1.814, *p* = 0.070) and disgust (*z* = 1.728, *p* = 0.084). For discrimination between MCI and FTD, negative emotions had a marginally larger AUC compared to positive emotions (*z* = 1.707, *p* = 0.088). For discrimination between AD and FTD, there was no significant difference between scores.

### Voxel-Based Morphometry Correlations with Facial Emotion Recognition Scores

**FIGURE 4:**
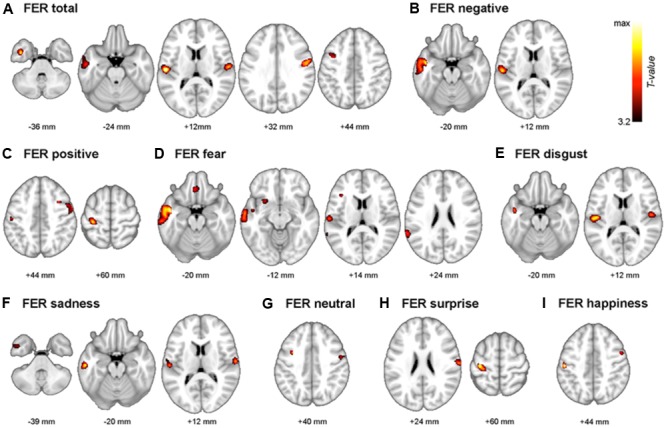
The multimodal system of FER. Gray matter regions that are associated with FER are depicted. **(A)** FER total; mean of all 7 emotions, **(B)** FER negative; mean of fear, anger, disgust, and sadness. **(C)** FER positive; mean of surprise and happiness. **(D)** fear, **(E)** disgust, **(F)** sadness, **(G)** neutral, **(H)** surprise, and **(I)** happiness. Values beneath the images indicate positions of Axial sections from the Montreal Neurological Institute average brain template in neurological convention (left to right) and displayed at *p* < 0.001, uncorrected for multiple comparisons and minimal size of spatially continuous cluster greater than 50 voxels.

The neuroanatomical correlates of FER scores from the voxel-based multiple regression analysis are summarized in **Table 4**. Gray matter volume in the temporal gyrus was positively correlated with the negative emotion recognition score, whereas the positive emotion recognition score showed a positive correlation with gray matter volume in the pre- and postcentral gyrus. Fear showed significant correlations with superior and middle temporal gyrus volume, including the temporal pole and insula, gyrus lectus and left inferior frontal gyrus. Disgust showed positive correlations with the left and right rolandic operculum including insula, and middle and inferior temporal gyrus volume. Sadness also showed a positive correlation with the volume of temporal regions (i.e., middle and inferior temporal gyrus), rolandic operculum and postcentral gyrus. Surprise and happiness showed positive associations with pre- and postcentral gyrus volume. **Figure 4** depicts the brain regions that positively correlated with each emotion.

**Table 4:** Brain regions showing positive correlations between facial emotion recognition scores and gray matter volume.

Measure	Region	BA	L/R	*T*	*k*	Peak MNI coordinate		
						*x*	*y*	*z*
Total	Middle frontal gyrus	8	L	3.715	237	–37.5	6.0	46.5
	Precentral gyrus	6	R	4.043	1508	52.5	–7.5	28.5
	Superior temporal gyrus	22	L	4.491	754	–54.0	–19.5	12.0
	Middle temporal gyrus	21	L	3.764	440	–54.0	–9.0	–22.5
	Inferior temporal gyrus	20	L	4.227	258	–40.5	–1.5	–36.0
Negative	Superior temporal gyrus	43	L	4.347	665	–55.5	–18.0	12.0
	Middle/Inferior temporal gyrus	20/21	L	4.783	2616	–42.0	0	–37.5
Positive	Pre/post central gyrus	3/6	R	4.191	1727	57.0	–10.5	30.0
		9	R	3.663	53	33	10.5	43.5
		4	L	4.260	596	–34.5	–28.5	54.0
		3	L	3.508	52	–49.5	–19.5	43.5
**Each emotion**								
Fear	Gyrus rectus	11	L	3.789	244	–3.0	34.5	–18.0
	Supramarginal gyrus	40	L	3.899	598	–63.0	–46.5	22.5
	Superior temporal gyrus	42	L	4.243	291	–60.0	–18.0	12.0
	Middle temporal gyrus	21	L	4.819	4080	–51.0	–4.5	–24.0
	Insula		L	3.680	136	–27.0	12.0	–12.0
				3.593	76	–37.5	21	1.5
	Inferior frontal gyrus	13	L	3.801	59	–37.5	21	15
Anger	None							
Disgust	Middle temporal gyrus	21	L	3.989	185	–45.0	–4.5	–19.5
	Inferior temporal gyrus	21	L	4.585	245	–43.5	–1.5	–37.5
	Rolandic operculum	13	L	4.304	929	–48.0	–16.5	10.5
			R	3.896	320	51.0	–12.0	12.0
Sadness	Postcentral gyrus	42	L	3.963	230	–57.0	–19.5	13.5
	Rolandic operculum	42/43	R	3.883	200	63.0	–12.0	12.0
	Middle temporal gyrus	21	L	3.455	175	–45.0	1.5	–39.0
	Inferior temporal gyrus	20	L	4.393	621	–57.0	–15.0	–22.5
Neutral	Middle frontal gyrus	9	L	3.963	140	–36.0	6.0	43.5
	Precentral gyrus	6	R	3.405	117	52.5	–3.0	39.0
	Cerebellum		L	3.745	263	–37.5	–87.0	–31.5
Surprise	Precentral gyrus	4	L	4.053	484	–34.5	–24.0	57.0
	Postcentral gyrus	4	R	3.754	637	63.0	–1.5	34.5
Happiness	Precentral gyrus	6	R	3.462	119	51.0	3.0	42.0
		6	R	3.734	131	52.5	–7.5	21.0
	Postcentral gyrus	3	L	3.788	155	–51.0	–19.5	43.5

*MNI, Montreal Neurological Institute.*

*Total, mean of all 7 emotional scores; negative, mean of fear, anger, disgust, and sadness; positive, mean of surprise and happiness.*

**FIGURE 5:**
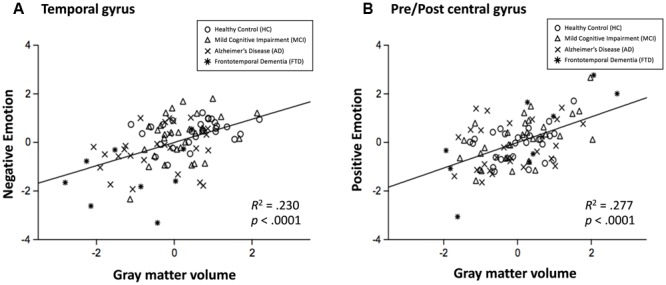
Scatterplots for the relationship between negative emotion recognition and temporal gyrus volume **(A)**, and relationship between positive emotion recognition with pre/postcentral gyrus volume **(B)**. Circle = HC, triangle = MCI, x mark = AD, and asterisk = FTD.

As a further analysis, associations between negative emotion recognition and the temporal gyrus volume and associations between positive emotion recognition and pre/postcentral gyrus volume were examined. Scatter plots are presented in **Figure 5**. The *R*^2^ was 0.230 (*p* < 0.001) in the association between negative emotion recognition and temporal gyrus volume, and 0.277 (*p* < 0.001) in the association between positive emotion recognition and pre/postcentral gyrus volume. The results of correlations in each region of interest (i.e., superior temporal gyrus, middle/inferior temporal gyrus, precentral gyrus, and postcentral gyrus) with negative and positive emotion recognition are presented in **Supplementary Figures S4** and **S5**, respectively.

## Discussion

In the present study, we investigated how FER scores differ among HC, and individuals with MCI, AD, and FTD. Furthermore, structural changes in gray mater structures that are related to FER performance of each emotion were demonstrated using automated methods of structural brain analysis. The extent of differential deficits in MCI, AD, and FTD on processing specific emotions has been clarified. The current study showed profiles of FER in older adults (i.e., HC, MCI, AD, and FTD) and find neural correlates of FER, in particular the correlation between recognizing specific emotions and the gray matter volume of specific brain regions.

### Differences in Performance across Groups and Contents of Emotions

#### Differences in Performance across Groups

Patients with FTD had the lowest total and negative scores, as well as the lowest scores for fear, anger, disgust, sadness, and neutral faces. These deficits were statistically significant for the mean negative score and fear when compared to HCs and patients with MCI and AD. These results corroborate the hypothesis that FTD would lead to the lowest FER performance when compared to HCs and patients with AD or MCI in older Korean participants. The FER impairments may be one reason for the high level of caregiver stress and depression in patients with FTD (Mioshi et al., 2009). Furthermore, although both patients with FTD and those with AD had similar impairments in cognitive functions (i.e., MMSE and CERAD), those with FTD had lower score for the negative emotions and fear. These results may be attributed to the different facets of cognitive impairments associated with emotional processing. For instance, impairments in emotional recognition are related to either verbal or spatial abilities in patients with AD (Cadieux and Greve, 1997). Memory encoding and retrieval for unpleasant stimuli have deficits in AD (Hamann et al., 2000). In contrast, it appears that emotional recognition deficits in FTD are more related to automatic processes, such as leading attention toward emotional stimuli, emotional arousal, or intuitive mechanisms (for a review, see Kumfor and Piguet, 2012).

Patients with AD had impaired FER in total mean score, mean score for negative emotions, and sadness when compared to HCs. These results were consistent with some and inconsistent with other previous studies. Patients with mild AD were impaired in the FER (i.e., fearful, angry, sad, disgusted, and happy) when compared to HCs in the Penn Emotion Recognition task (Spoletini et al., 2008). Fear, sadness, disgust, and happiness recognition were impaired in patients with AD when compared to HCs regardless of intensity, although the difference between the AD and HC groups was not significant in low-intensity anger stimuli. Disgust and fear, but not anger, sadness, and joy, were impaired in patients with AD when compared to HCs (Wiechetek et al., 2011). In contrast, another study reported that patients with AD were not impaired in recognizing facial emotions, including fear, anger, disgust, sadness, surprise, and happiness (Burnham and Hogervorst, 2004). These insufficient and inconsistent results hinder any firm conclusions regarding FER performance in patients with AD. It should be noted that not all of the participants had the same severity of disease. Thus, differences in cognitive impairments, such as verbal deficits or visuospatial functions in the patients with AD might have led to the inconsistency (Cadieux and Greve, 1997). Furthermore, as shown in the study by Wiechetek et al. (2011), differences in stimuli may also lead to inconsistency. Further studies should be carried out to clarify these inconsistent findings.

Patients with MCI had no statistically significant differences with HCs or patients with AD. Patients with FTD had lower performance than those with MCI for total score and negative, fear, anger, sadness, and neutral face stimuli. These results are contrary to those of a previous study reporting amnestic MCI deficits in emotion recognition (McCade et al., 2013a). The patients with MCI in this study had amnestic MCI. Patients with amnestic MCI in a previous study had similar performance on the FER to HCs and patients with AD (Bediou et al., 2009). Spoletini et al. (2008) also found that patients with amnestic MCI and HCs had no significant differences in total FER, but that the performances were different between patients with MCI and those with AD. As mentioned in the review of emotion recognition in MCI, this topic is in its “infancy” (McCade et al., 2012). It is difficult to draw any firm conclusions regarding this issue given the few studies in the literature. Heterogeneity within the MCI group or the methodologies used should be investigated in further studies.

#### Deficits in Negative Emotion Recognition

The principle finding of our study was that negative emotion recognition is impaired worst in FTD. Patients with FTD showed substantial deficits in the recognition of negative emotions, especially fear, and were distinguishable from patients with AD. In addition, the mean of each negative emotion (i.e., fear, anger, disgust, and sadness) can successfully differentiate sub-groups, while mean of each positive emotion (i.e., surprise and happiness) failed to distinguish among the groups. The negative emotions have higher sensitivity and specificity than positive emotions. The results further suggest that malfunction of inter-personal relations in FTD may be based on a failure to identify the negative mood of others, although there may be various factors and interrelated mechanisms that contribute to general interpersonal difficulties. In addition, it becomes obvious that using stimuli representing fear or negative emotions is crucial for the fast screening of FTD and estimating the severity of FTD on a clinical level.

These findings confirm previous reports that FTD patients show impaired recognition of negative facial emotions (i.e., fear, anger, disgust, and sadness) (Fernandez-Duque and Black, 2005). In recent studies that utilized the Ekman 60 faces test it was found that negative emotions (i.e., fear, anger, disgust, and sadness) showed higher effect sizes in distinguishing between HCs and patients with FTD (i.e., semantic dementia) than positive emotion recognition (i.e., surprise and happiness) (Hsieh et al., 2012b; Kumfor et al., 2013). In both the facial and musical emotion recognition test, FTD showed severe impairment in recognizing negative emotions than positive emotions. Temporal variants of FTD showed intact happiness recognition, whereas the ability to recognize sadness, anger, fear, and neutral was impaired (Rosen et al., 2004).

Negative emotions depicted in schematic faces can be automatically perceived by people, with negative emotions more effectively attracting focal attention than positive emotions (Eastwood et al., 2001). Recognizing unfavorable situations or negative facial stimuli has certain advantages in social outcomes and survival, thus this ability may innate. When this capability is impaired, as in FTD or AD, tremendous malfunction in social interactions occur. Deficits in the recognition of others’ facial emotion reflects impairments in the encoding and interpretation of social cues. Accurate detection of social cues is the first step of the social information processing mechanism (Crick and Dodge, 1994). If people are unable to detect social cues, following mechanisms, such as response decisions and behavioral enactments, would be inappropriate. Therefore, it would be an effective approach to alleviate malfunction in dementia patients by using teaching strategies to accurately detect social cues.

These results can be generalized to other sensory modalities and other objects. Previous studies have revealed that emotion recognition based on the vocal sensory modality is impaired in patients with FTD (Keane et al., 2002; Snowden et al., 2008) and detection of emotions based on static or dynamic body expression is impaired in patients with bvFTD (Van den Stock et al., 2015). Researchers have found deficits in negative emotion recognition in other stimuli, such as music (Omar et al., 2011; Hsieh et al., 2012b) or emotion words (Hsieh et al., 2012a). However, the profiles of FER performance among the patient group (i.e., FTD and AD) may be distinguished based on sensory modality (Koff et al., 1999). Hsieh et al. (2013) found that only negative FER was impaired in patients with bvFTD when compared to HCs, while positive FER was intact. In terms of emotion recognition of vocalization, both positive and negative emotion recognition were impaired in individuals with bvFTD. Patients with AD have impaired FER for fear, sadness, and disgust, but not for prosody or music (Drapeau et al., 2009). Therefore, further studies should be performed using various types of stimuli and participants.

#### Preservation in Positive Emotion Recognition

There were no differences among the HC, MCI, AD, and FTD in the current study. This result corroborates those of previous studies indicating that positive emotions are likely preserved in patients with neuropsychiatric disease (Rosen et al., 2002, 2006; Kessels et al., 2007; Calabria et al., 2009). However, Hsieh et al. (2012b) study demonstrated that positive emotions could differentiate FTD and HC groups, while the present study showed no significant difference. It is currently not clear why this difference in results is seen. It is possible that the inconsistency in the findings regarding differential ability to recognize positive emotions is due to sampling, methodological, or stimuli differences, which should be explored in the future. Since the intensity of facial expressions may be a moderating factor in distinguishing psychiatric groups (Phillips et al., 2010), we can attribute the inconsistency to the different levels of difficulty of the stimuli. The correct ratio of positive emotions in Hsieh et al. (2012b) study was over 90% in HCs. However, the corresponding value in the current study was 54%. It is possible that the task used in the previous study (Hsieh et al., 2012b) was affected by a ceiling effect. The presence of closed vs. open mouths would be a critical factor when determining the intensity of facial emotion expressions (Boucart et al., 2008). In fact, specific features of the face are more effectively used to process facial emotions (i.e., opened eyes in fear and opened mouth in happiness) (Schyns et al., 2009). Performances among groups may vary depending on the intensity of facial stimuli (Spoletini et al., 2008). The current study primarily followed the rules established by Ekman and Friesen (2003) to express facial emotion, and the intensity of each emotion was the same except surprise. Since the perceived intensity was different in surprise, it could affect to the results. Further studies should address the issue, and determine the most effective level of stimuli intensity.

### Multi-Model Neural System in Facial Emotion Recognition

Differences in the recognition of each emotion in patients with dementia and MCI have increased interest in the role of dissociable and non-overlapping neural substrates for the processing of specific facial emotions (Phan et al., 2002). In the neuropsychological literature, a substantial number of studies have revealed separate brain regions and distinct neural connections for decoding specific facial expressions (Sprengelmeyer et al., 1998; Blair et al., 1999; Fine and Blair, 2000; Calder et al., 2004). There may be many underlying reasons for this phenomenon (i.e., multi-model neural system in FER). One possible mechanism is based on the evolutionary perspective (Smith et al., 2005). Facial emotion expression in humans conveys information regarding the emotional state of the individual. It thus contains crucial data for social interactions. Based on the evolutionary framework, facial emotion expression has evolved to transmit emotion effectively, and the brain acts as a decoder for the interpretation of facial signals. In these processes, the brain can evolve to have a specialized neural network or structure to optimize its decoding performance (Phillips et al., 1997; Blair et al., 1999; Vuilleumier et al., 2003; Winston et al., 2003).

Both behavioral and neuronal results from the current study showed that the patterns of impairments in recognizing facial emotions are different according to the type of emotion. This result suggests that there is distinct processing in recognizing each emotion (Vytal and Hamann, 2010). On a theoretical level, our findings further establish the temporal and frontal regions as crucial in maintaining normal recognition of negative and positive emotions, respectively. Even though no one study can fully demonstrate the neural basis of human emotions, the results support the idea that the neural correlates of FER are distinct, depending on the contents of emotion. These findings support the notion that there may be distinct neural circuits in the processing of positive and negative emotions (Kim and Hamann, 2007; Mak et al., 2009).

The FER system in the brain is multimodal, as Ekman demonstrated (Ekman, 1999). There has been a substantial amount of evidence that recognition and expression of fear are related to the amygdala and surrounding regions (Adolphs et al., 1995, 1999). Furthermore, insula damage has been attributed with drastic impairment in the recognition and experience of disgust (Calder et al., 2000). Consistent with this, deficits in fear recognition correlate with gray matter volume of the amygdala and surrounding regions (i.e., gyrus rectus, superior, middle and inferior temporal gyrus, and insula). Deficits in disgust recognition have been correlated with gray matter volume in the insula and surrounding regions (i.e., Rolandic operculum, and middle and inferior temporal gyrus). Therefore, the results of this study provide conclusive evidence that recognizing fear and disgust is closely related to the amygdala, insula, and surrounding regions.

In contrast to fear and disgust, anger showed no significant correlations. In previous studies, recognition of anger was related with damage to the ventral striatum (Calder et al., 2004) or middle and superior temporal gyrus (Kumfor et al., 2013). Furthermore, recognizing sadness is known to correlate with the left subcallosal cortex (Kumfor et al., 2013) or left orbitofrontal and mid-dorsolateral frontal cortex (Khalfa et al., 2005), while the present study showed a positive correlation with postcentral gyrus and temporal regions. This inconclusive evidence may support the hypothesis that the brain regions that reflect recognition of anger or sadness are relatively variable. The neural correlates related with the recognition of these facial emotions may be distinct depending on contextual factors such as the characteristics of participants or materials of tasks. For instance, there are gender differences in the neural processing of sadness recognition (Lee T.M. et al., 2002). Therefore, more sophisticatedly designed experiments should be performed to find neural substrates of FER in anger and sadness.

Surprise and happiness showed positive correlations in the pre- and postcentral gyrus. Even though there are only a small number of brain studies, and there has been no consistent conclusion about the brain activation of happy faces (Posamentier and Abdi, 2003), frontal regions are generally considered to be responsible for processing positive emotions. For instance, the ability to detect happiness is positively correlated with the intactness of dorsal and/or lateral prefrontal cortices (Heberlein et al., 2008). In the fMRI study, the medial frontal cortex and right supramarginal gyrus were activated during the processing of happy faces (Phillips et al., 1998). A positron emission tomography (PET) study revealed that the presentation of happy faces was related to activation in the left ventral prefrontal cortex (Dolan et al., 1996). These results support the idea that frontal regions are responsible for processing positive faces. Furthermore, previous studies reveal that participants find it easier to regulate positive emotions than negative emotions (Mak et al., 2009). In the present study, the positive emotional processing was related to dorsal regions (i.e., pre/post central gyrus) that are considered more important for the regulation of emotion than ventral parts (Phillips et al., 2003), thus the regulation of positive emotions can be more manageable. However, there are inconsistencies in the frontal regions that correspond to processing positive emotions, thus more evidence will be needed (e.g., Habel et al., 2005).

### Limitations

There are several limitations in this study. First, we collapsed data across FTD subtypes. There were both patients with bvFTD (*n* = 8) and those with SD (*n* = 5) in this study. Previous studies investigated differences in FER (Keane et al., 2002; Rosen et al., 2004; Kumfor et al., 2011) and brain atrophy (Garibotto et al., 2011; Agosta et al., 2012) depending on FTD subtypes (Kumfor et al., 2013), thus further study should be performed to find what emotions have large effect sizes in the ability to distinguish FTD subtypes. Furthermore, the number of FTD was relatively small (i.e., 13). There were practical difficulties that the FTD patients were not easy to recruit in the experiments because they are generally uncooperative and HC and MCI were more to visit the two centers (i.e., SMG-SNU Boramae Medical Center and Dongjak-Gu Center for Dementia). It is possible that the small number of FTD may affect to the power and generalization of the statistical results. However, the effects size of facial-emotion recognition in the statistics distinguishing FTD with NC or AD (e.g., *d* = 1.23 from the study of Bora et al., 2016) was large (Cohen, 1992). And G^∗^power analysis showed that we can get the power of 0.82 when we had 22 NC (or AD) and 8 FTD in the mean comparison (two tails, large effect size, 0.05 alpha, allocation ration N2/N1 was 3). Second, only patients with amnestic MCI participated in the current study. Even though there has been a previous study reporting that only patients with amnestic MCI have emotional recognition deficits (McCade et al., 2013a), we were unable to find differences in performance between the patients with MCI and HCs or patients with AD. Furthermore, impairment of anger recognition in patients with non-amnestic MCI was negatively correlated with basic and instrumental activities of daily living (McCade et al., 2013a). Therefore, including both patients with amnestic MCI and those with non-amnestic MCI in further studies would lead to a crucial understanding of their FER and caregiver burden. Third, the FER test is somewhat different from the real processing of social emotional functions in everyday lives. People do not need to verbally label emotion, and we may have implicit and non-conscious mechanisms to process others’ facial emotions (Whalen et al., 2004). Therefore, there remains a need to develop tools that are more ecologically validated and represent real processing of emotional recognition in our daily routines. One possible option is to utilize animated real-life social scenarios, a procedure that has been used to assess empathy deficits in FTD (Baez et al., 2014).

## Conclusion

Despite these limitations, the current study addresses an important area of interest in FER. Data examining the performances and neuroanatomical characteristics of HCs and patients with MCI, AD, and FTD have supported multimodal system theories of emotion processing. The mean score of negative emotion recognition (i.e., fear, anger, disgust, and sadness) showed the largest effect size to distinguish HC, MCI, AD, and FTD. The negative emotion score was correlated with gray matter volume in temporal regions, whereas positive emotion recognition was correlated with frontal regions. The results help to develop theoretical models in affective cognitive neuroscience of multimodal systems at a gray matter level. Furthermore, testing negative emotion recognition would help in reducing the time and cost of understanding FER in clinics.

## Author Contributions

Substantial contributions to the conception or design of the work: J-YL, J-HY, and BS. The acquisition, analysis, or interpretation of data for the work: SS, YK, BS, H-JP, SP, and J-YL. Drafting the work or revising it critically for important intellectual content: SP, TK, and J-YL. Final approval of the version to be published: SP and J-YL.

## Conflict of Interest Statement

The authors declare that the research was conducted in the absence of any commercial or financial relationships that could be construed as a potential conflict of interest.

## Footnotes

1 http://www.mathworks.com

## Supplementary Material

FIGURE S1 Monitor screen of a practice trial. An example facial item and seven options were presented before the start of the actual test. All options were presented in Korean.

FIGURE S2 Examples of pictorial stimuli in the facial emotion recognition (FER) test. **(A)** Fear, **(B)** anger, **(C)** disgust, **(D)** sadness, **(E)** neutral, **(F)** surprise, and **(G)** happiness.

FIGURE S3 Gray matter volume comparison between the healthy control (HC), patient with mild cognitive impairment (MCI), patient with Alzheimer’s disease (AD), and patient with frontotemporal dementia (FTD). **(A)** Gray matter volume reductions were detected in the left inferior temporal gyrus, bilateral parahippocampal gyri, and left superior temporal gyrus in the patient with MCI when compared to the HC. **(B)** Bilateral putamen extending to the parahippocampal gyrus, superior and middle temporal gyri, middle occipital gyrus, and the cingulate showed gray matter volume reductions in the patient with AD. **(C)** Volume of the bilateral putamen extending to the parahippocampal gyrus and middle temporal gyrus was decreased in the patient with FTD. There were no between-group differences in total intracranial or gray matter volumes, except for total gray matter volume between the HC and the patient with AD (smaller in the patient with AD; *p* < 0.032, using two-sample *t*-test).

FIGURE S4 Scatterplots for the relationship between negative emotion recognition and superior temporal gyrus volume **(A)** and middle/inferior temporal gyrus volume **(B)**. Circle = healthy control (HC), triangle = mild cognitive impairment (MCI), x mark = Alzheimer’s disease (AD), asterisk = frontotemporal dementia (FTD).

FIGURE S5 Scatterplots for the relationship between positive emotion recognition and right precentral gyrus region volume of MNI coordinate 33, 10.5, 43.5 **(A)**, 57, -10.5, 30 **(B)**, left postcentral gyrus volume (-49.5, -19.5, 43.5) **(C)**, and right precentral gyrus volume (-34.5, -28, 54) **(D)**. Circle = HC, triangle = MCI, x mark = AD, asterisk = FTD.
