# Corrigendum: Behavioral and Neuroimaging Evidence for Facial Emotion Recognition in Elderly Korean Adults with Mild Cognitive Impairment, Alzheimer's Disease, and Frontotemporal Dementia

**DOI:** 10.3389/fnagi.2019.00091

**Published:** 2019-04-24

**Authors:** Soowon Park, Taehoon Kim, Seong A. Shin, Yu Kyeong Kim, Bo Kyung Sohn, Hyeon-Ju Park, Jung-Hae Youn, Jun-Young Lee

**Affiliations:** ^1^Department of Education, Sejong University, Seoul, South Korea; ^2^Department of Psychiatry, Neuroscience Research Institute, Seoul National University and SMG-SNU Boramae Medical Center, Seoul, South Korea; ^3^Department of Biomedical Sciences, Seoul National University, Seoul, South Korea; ^4^Department of Nuclear Medicine, SMG-SNU Boramae Medical Center, Seoul, South Korea; ^5^Department of Psychiatry, Inje Univiersity Sanggye Paik Hospital, Seoul, South Korea; ^6^Graduate School of Clinical Counseling Psychology, CHA University, Pocheon, South Korea

**Keywords:** facial emotion recognition, mild cognitive impairment, Alzheimer's disease, frontotemporal dementia, voxel-based morphometry

In the original article, we neglected to include the funder of the “Basic Science Research Program, through the National Research Foundation of Korea (NRF) funded by the Ministry of Education (No. NRF-2017R1D1A1A02018479).”

A correction has therefore been made the **Funding** statement:

“This study was conducted in Seoul, South Korea. This study was supported by a grant of the Korean Health Technology R&D Project, Ministry of Health and Welfare, South Korea (HI07C0001), as well as a grant from the Basic Science Research Program, through the National Research Foundation of Korea (NRF) funded by the Ministry of Education (No. NRF-2017R1D1A1A02018479).”

The authors apologize for this error and state that this does not change the scientific conclusions of the article in any way. The original article has been updated.

