# Supplementary figures and images for: Behavioral and Neuroimaging Evidence for Facial Emotion Recognition in Elderly Korean Adults with Mild Cognitive Impairment, Alzheimer’s Disease, and Frontotemporal Dementia
Source: Front Aging Neurosci. 2017 Nov 30;9:389. doi: 10.3389/fnagi.2017.00389 (PMC5714934; doi:10.3389/fnagi.2017.00389)

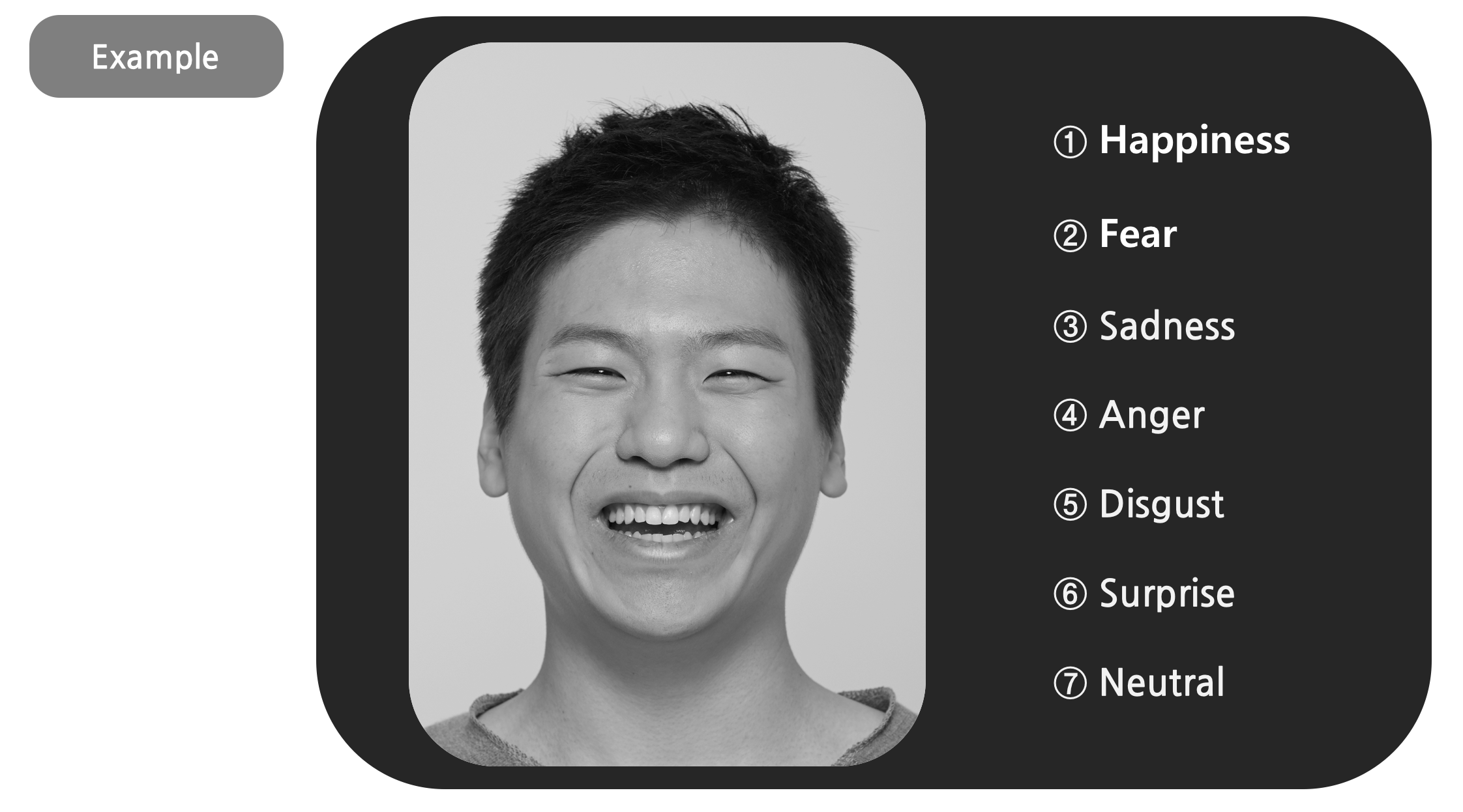

Supplement: FIGURE S1 — Monitor screen of a practice trial. An example facial item and seven options were presented before the start of the actual test. All options were presented in Korean. [file Image_1.png]

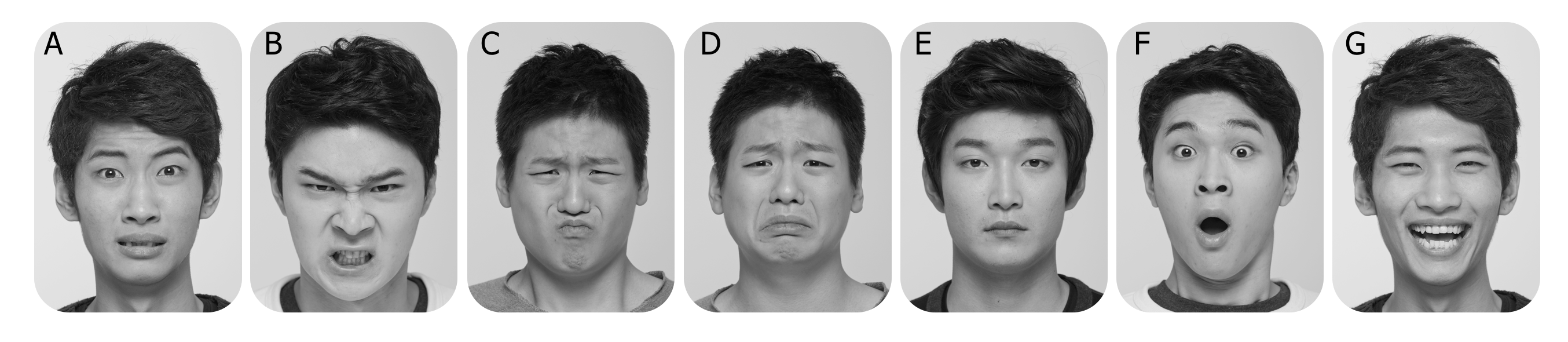

Supplement: FIGURE S2 — Examples of pictorial stimuli in the facial emotion recognition (FER) test. (A) Fear, (B) anger, (C) disgust, (D) sadness, (E) neutral, (F) surprise, and (G) happiness. [file Image_2.png]

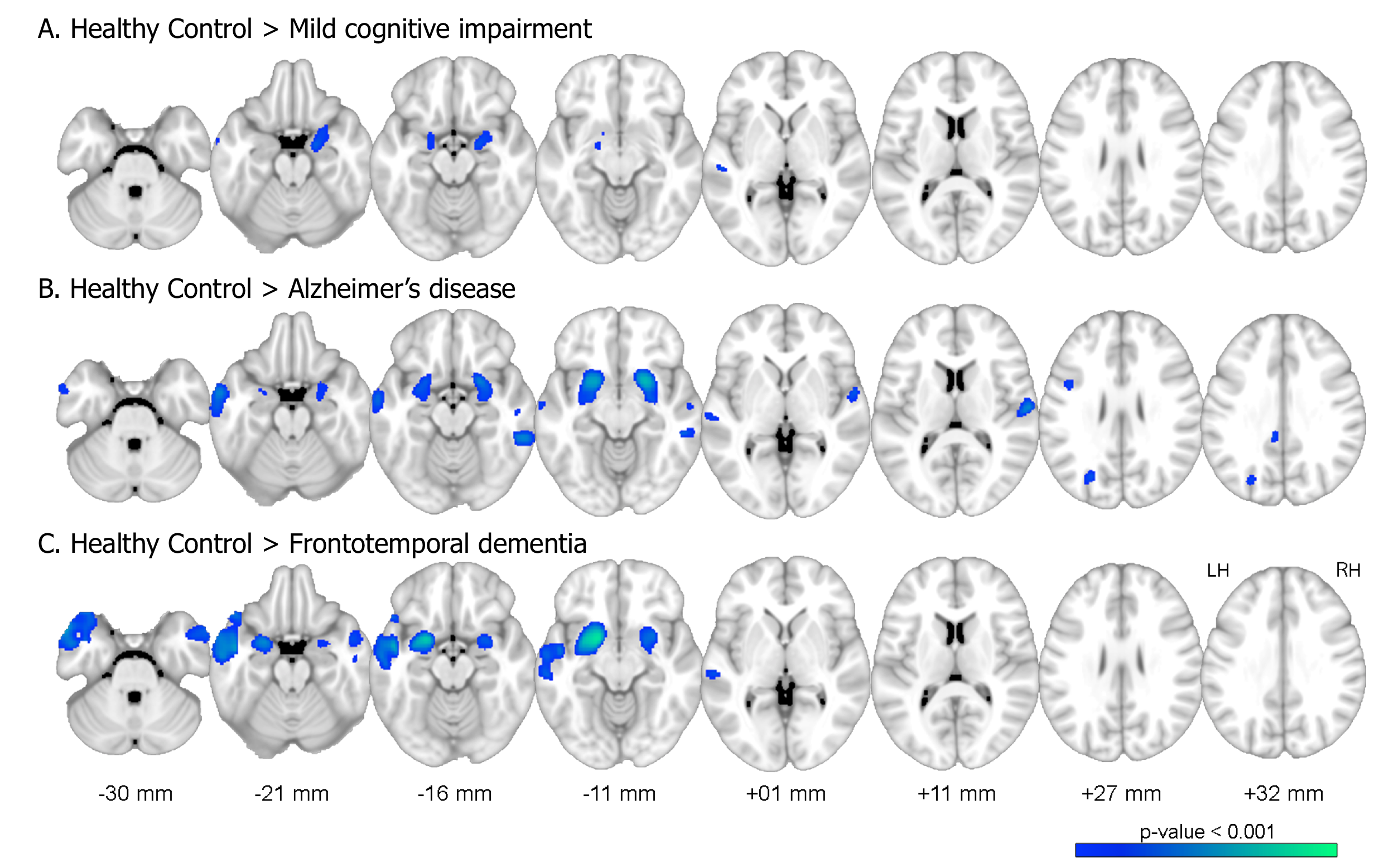

Supplement: FIGURE S3 — Gray matter volume comparison between the healthy control (HC), patient with mild cognitive impairment (MCI), patient with Alzheimer’s disease (AD), and patient with frontotemporal dementia (FTD). (A) Gray matter volume reductions were detected in the left inferior temporal gyrus, bilateral parahippocampal gyri, and left superior temporal gyrus in the patient with MCI when compared to the HC. (B) Bilateral putamen extending to the parahippocampal gyrus, superior and middle temporal gyri, middle occipital gyrus, and the cingulate showed gray matter volume reductions in the patient with AD. (C) Volume of the bilateral putamen extending to the parahippocampal gyrus and middle temporal gyrus was decreased in the patient with FTD. There were no between-group differences in total intracranial or gray matter volumes, except for total gray matter volume between the HC and the patient with AD (smaller in the patient with AD; p < 0.032, using two-sample t-test). [file Image_3.png]

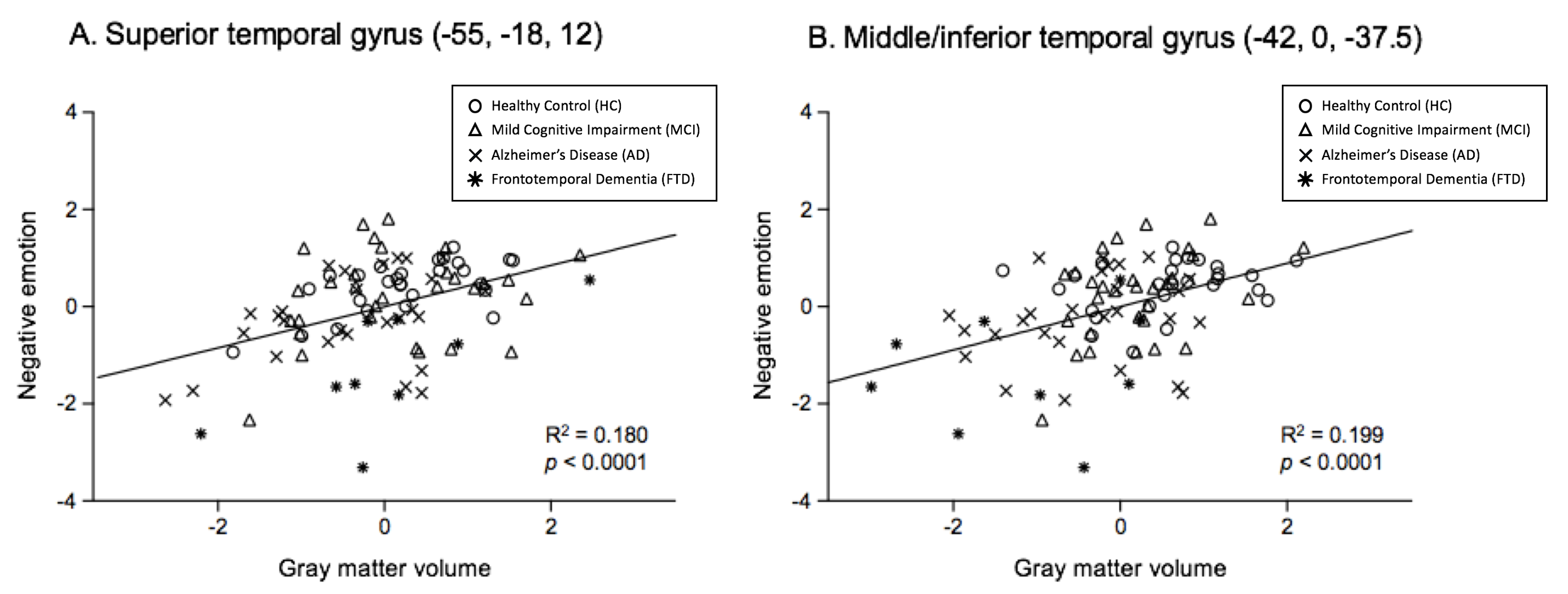

Supplement: FIGURE S4 — Scatterplots for the relationship between negative emotion recognition and superior temporal gyrus volume (A) and middle/inferior temporal gyrus volume (B). Circle = healthy control (HC), triangle = mild cognitive impairment (MCI), x mark = Alzheimer’s disease (AD), asterisk = frontotemporal dementia (FTD). [file Image_4.png]

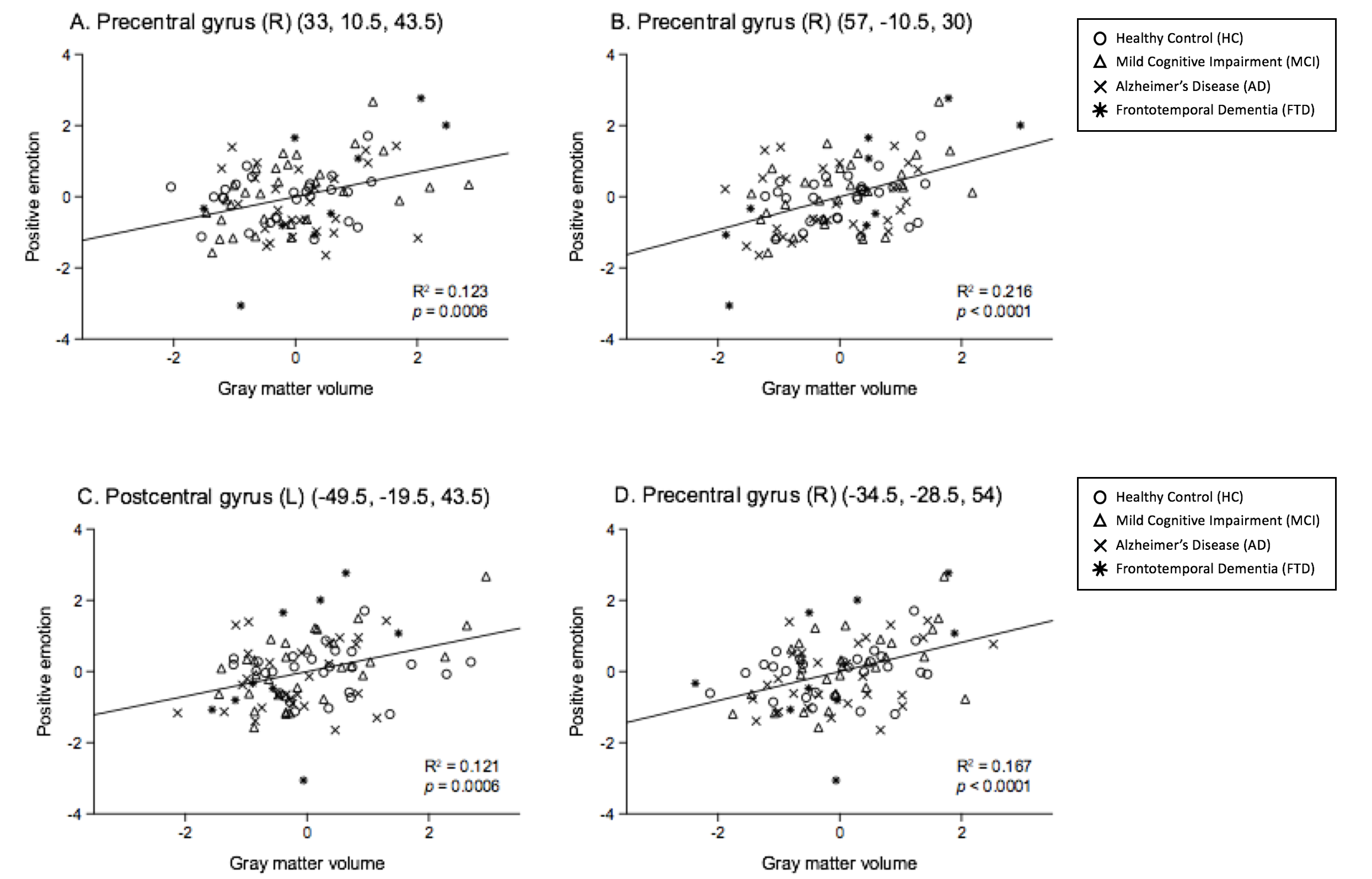

Supplement: FIGURE S5 — Scatterplots for the relationship between positive emotion recognition and right precentral gyrus region volume of MNI coordinate 33, 10.5, 43.5 (A), 57, -10.5, 30 (B), left postcentral gyrus volume (-49.5, -19.5, 43.5) (C), and right precentral gyrus volume (-34.5, -28, 54) (D). Circle = HC, triangle = MCI, x mark = AD, asterisk = FTD. [file Image_5.png]
